# The difference between the Connor–Davidson Resilience Scale and the Brief Resilience Scale when assessing resilience: confirmatory factor analysis and predictive effects

**DOI:** 10.1017/gmh.2022.38

**Published:** 2022-07-19

**Authors:** Yun-Ci Ye, Chia-Huei Wu, Tzu-Yun Huang, Cheng-Ta Yang

**Affiliations:** 1 Department of Somatics and Sport Leisure Industry, College of Humanities, National Taitung University, Taitung, Taiwan; 2 Management Department, Leeds University Business School, University of Leeds, Leeds, UK; 3 Department of Medical Research, China Medical University Hospital, China Medical University, Taichung, Taiwan; 4 Department of Psychology, National Cheng Kung University, Tainan, Taiwan; 5 Graduate Institute of Mind, Brain and Consciousness, Taipei Medical University, Taipei, Taiwan

**Keywords:** Brief Resilience Scale, confirmatory factor analysis, Connor–Davidson Resilience Scale, depression, life satisfaction, resilience

## Abstract

**Background:**

The Connor–Davidson Resilience Scale (CD-RISC) and the Brief Resilience Scale (BRS) are two scales widely used to measure resilience. Although both scales seek to assess an individual's ability to recover from and adapt to disruptions or stressful events, they can capture different aspects of resilience. While the CD-RISC focuses on resources that can help individuals to recover from and adapt to disruptions or stressful events, the BRS directly measures one's ability to bounce back or be resilient. The aim of this study is to better understand resilience through empirically examining the differences between the CD-RISC and the BRS.

**Method:**

Samples (a pooled sample *N* = 448 and two subsamples *N* = 202 and 246) consisting of undergraduate students from Taiwan were used. Confirmatory factor analysis (CFA) was performed to examine the relationship between the CD-RISC and BRS. Regression analysis was conducted to examine predictive effects of the CD-RISC and BRS on depression and life satisfaction.

**Result:**

The results of CFA using different samples consistently show that the CD-RISC and the BRS are highly correlated but still distinct. The results of regression analyses using different samples also consistently show that the CD-RISC and the BRS have unique predictive effects regarding depression and life satisfaction.

**Conclusions:**

The research findings suggest that the CD-RISC and the BRS capture different aspects of resilience. For future research on resilience, researchers should pay closer attention to the differences between these scales and choose the one that most closely fits their research purpose.

## Introduction

Individual resilience has been recognized as an important topic in behavioral and medical science (Masten, 2001; Charney, 2004). Over the past decade, resilience has been conceptualized from different angles, and different tools have been developed to assess resilience in different ways (Pangallo *et al*., 2015). Among the available tools to measure resilience, the Connor–Davidson Resilience Scale (CD-RISC) (Connor and Davidson, 2003) and the Brief Resilience Scale (BRS) (Smith *et al*., 2008) are two widely used scales. Although both tools seek to assess an individual's ability to recover from and adapt to disruptions or stressful events, they are different in several ways.

The fundamental difference between the CD-RISC and the BRS is the conceptualization of resilience. The CD-RISC was originally designed to serve as a clinical measurement for posttraumatic stress disorder patients and was established on the beginning point of biopsychospiritual balance (or homeostasis), the ideal status of mental well-being. Thus, resilience is conceptualized as a multidimensional concept in the CD-RISC (Connor and Davidson, 2003), incorporating resources into different aspects that can help individuals to achieve biopsychospiritual balance. Stated alternatively, the CD-RISC was established on a resource-based perspective of coping with stress (Hobfoll, 2011) and captures resilience as an accessible resource that could enhance the ability to manage stress and adversity. Accordingly, the CD-RISC includes not only items about an individual's internal resources, such as personal competence, but also items regarding accessibility to social resources (i.e. close and secure relationships) and environmental resources (i.e. know where to turn for help). Specifically, this scale assesses resilience by examining five dimensions: (1) personal competence; (2) tolerance of negative affect and the strengthening effects of stress; (3) positive acceptance of change; (4) a sense of control; and (5) spiritual influence.

In contrast, resilience is conceptualized as a unidimensional concept in the BRS. As indicated by Smith *et al*. (2008), the authors sought to ‘clarify the study of resilience by presenting a scale for assessing the original and most basic meaning of the word resilience’ and developed the BRS to ‘assess the ability to bounce back or recover from stress’. The BRS therefore focuses on an individual's ability to bounce back and does not consider external resources. All of the items on the BRS start with and revolve around the ‘self’ or the belief in one's ability to bounce back (i.e. I tend to bounce back quickly after difficult times). Conceptually, the BRS reflects one's belief in recovering from stress, in line with the idea of social cognitive theory (Bandura, 1999), in which a strong sense of self-efficacy can motivate individuals to undertake action to achieve a specific goal effectively.

We argue that both conceptualizations of resilience are equally important to one's well-being, which is the case because, to recover from adversity, one will not only need to have his or her own psychological resources and external resources to cope with the situations (Hobfoll, 2011; Hilliard *et al*., 2015) but also need to have a strong belief that he or she is capable of bouncing back from difficult situations (Smith *et al*., 2008). In addition, the two conceptualizations of resilience also bring different lenses to understanding the implications of resilience to one's well-being. Specifically, following the resource-based perspective of stress coping (Hobfoll, 2011), managing stress can deplete one's resources, motivating an individual to maintain and pursue resources to sustain the process of combatting stress and bounce back from adversity. Resource-based resilience is thus important to one's well-being because the more resources that an individual has, the greater that the chance is that s/he can cope with an unfavorable situation without being depleted, preventing the individual from having mental health problems, such as depression (Hobfoll, 2011). Having more resources also helps individuals to close the gap between what they are and what they seek to achieve, contributing to one's well-being, such as greater life satisfaction. In contrast, social cognitive theory (Bandura, 1999) emphasizes the importance of a sense of personal agency and suggests that a strong belief in one's agency in undertaking actions to approach a specific goal can sustain one's effort and keep an individual persistent in goal achievement. Following this theory, we argue that belief-based resilience can contribute to individual well-being because it keeps an individual focused on goal achievement instead of being depressed and frustrated by the current situation. Belief-based resilience can also motivate individuals to maximize their efforts for goal striving and thus promote life satisfaction by reducing goal discrepancy. As such, theoretically, we expect that resource-based and belief-based resilience will contribute to individual well-being for different reasons, suggesting that the CD-RISC and BRS could make independent contributions to individual well-being.

Nevertheless, to our knowledge, there has been no empirical work demonstrating their differences. While a methodological review of resilience measurement scales, including the CD-RISC and BRS, was performed (Windle *et al*., 2011), the differences between the CD-RISC and BRS have not been discussed. Most empirical studies of resilience have only applied one of the scales as the indicator of resilience. For instance, Abolghasemi and Varaniyab (2010), Chow and Choi (2019), and Mcdermott *et al*. (2020) used the BRS solely as the indicator of resilience, whereas Bajaj and Pande (2016), Chue and Cheung (2021) and Wingo *et al*. (2010) used the CD-RISC alone to assess resilience when they investigated the association between resilience and well-being indicators, such as depression, life satisfaction, and physical activity levels. Whether the CD-RISC and BRS have unique predictive effects on well-being indicators is unknown.

The aim of this study is to examine the differences between the CD-RISC and the BRS in two steps. First, we perform confirmatory factor analysis (CFA) to examine the relationships between the two measurements. To date, studies have only reported their simple correlations (Smith *et al*., 2008; Rodríguez-Rey *et al*., 2016) without examining their factor structures simultaneously. Without correcting measurement errors and controlling for potential wording effects due to the inclusion of negative-worded items (Wu, 2008) on the BRS, the correlation between the CD-RISC and the BRS cannot be properly assessed. We thus aim to examine the relationship between the CD-RISC and the BRS based on the latent factors extracted from each measurement. Second, to examine our hypothesis that the CD-RISC and the BRS capture different aspects of resilience that are important for well-being, we use the CD-RISC and the BRS to predict depression and life satisfaction – a negative indicator and a positive indicator of well-being, respectively. We expect that both the CD-RISC and the BRS will have significant predictive effects on depression and life satisfaction.

We use samples consisting of university students in this study because resilience has been found to be a critical factor in university students' well-being, using either the CD-RISC or the BRS as a measure of resilience. For example, using the CD-RISC as a measure of resilience, Chue and Cheung (2021) reported that resilience, measured by the CD-RISC, helps to prevent burnout and promote mental health among college students. Hartley (2011) also reported a strong correlation between resilience factors on the CD-RISC and mental health in college students. Collen and Onan (2021) reported a positive correlation between resilience, measured by the CD-RISC, and psychological well-being in university students. Using the BRS as a measure of resilience, Chow and Choi (2019) reported that resilience, measured by the BRS, predicted the mental health status of college freshmen. Mcdermott *et al*. (2020) found an association between resilience, measured by the BRS, and well-being among college nursing students. While much research has been performed with university student populations using either the CR-RISC or the BRS to assess resilience, we believe that we should clarify the differences between these two measurements to better understand the resilience of university students and determine how to support and promote the resilience of the population.

## Materials and methods

### Participants and procedures

We recruited participants consisting of undergraduate students in 2019 and 2020. We recruited participants from among students in introductory psychology courses at National Cheng Kung University. For psychology students, participating in psychological experiments is one of the requirements for introductory psychology courses, providing students with research experiences by having them participate in studies. Every year, students in courses can enroll in different psychological experiments voluntarily based on their interests and receive course credit for their participation, which is the procedure for how we recruited undergraduate students from psychology courses. We also recruited undergraduate students online to approach those who were not in introductory psychology courses. These participants were reimbursed with 150 New Taiwan dollars per hour for their participation. Sample 1 included 202 participants in 2019. The mean age of the participants was 20.19 years old (s.d. = 1.31), and 63.4% were female. Sample 2 included 246 participants in 2020. The mean age was 19.67 years old (s.d. = 1.25), and most of the participants were male (61.2%). Each time we recruited approximately 200 participants based on the rule of thumb for a sample size performing structural equation models (i.e. CFA in this study) (Bentler and Chou, 1987; Kline, 2011). To have a large sample size for the subsequent analysis, we merged the data together and used the sample with 448 participants in the following report. We also performed the same analysis using the two samples separately for cross-validation.

#### The Brief Resilience Scale (BRS)

The BRS consists of six items (Smith *et al*., 2008). Each item was rated on a five-point scale ranging from 1 = ‘strongly disagree’ to 5 = ‘strongly agree’. We used the traditional Chinese version validated by Tu *et al*. (2017). An average score is used to indicate the level of resilience. The Cronbach's *α* coefficients were 0.76, 0.76, and 0.79 in the pooled sample and in samples 1 and 2, respectively.

#### The Connor–Davidson Resilience Scale (CD-RISC)

The CD-RISC evaluates the psychological trait of resilience (Connor and Davidson, 2003). The questionnaire contains 25 items encompassing five dimensions: personal competence, trust, positive acceptance, control, and spiritual influence. Items were rated on a five-point scale ranging from not true at all (0) to true nearly all of the time (4). A total score was used to indicate greater resilience. We used a traditional Chinese version validated by Wang *et al*. (2017). The Cronbach's *α* coefficients were 0.93, 0.93, and 0.91 in the pooled sample and in samples 1 and 2, respectively.

#### The Beck Depression Inventory Second Edition (BDI-II)

The BDI-II was used to assess depression (Beck *et al*., 1996). The scale consists of 21 items calculated on a four-point scale from 0 to 3. After totaling all of the items, the total score ranged from 0 to 63, with higher scores indicating a higher level of depression. We used a traditional Chinese version. Its reliability and validity were reported by Lu *et al*. (2002). The Cronbach's *α* coefficients were 0.92, 0.92, and 0.88 in the pooled sample and in samples 1 and 2, respectively.

#### The Satisfaction With Life Scale (SWLS)

The SWLS measures an individual's cognitive perception of subjective well-being (Diener *et al*., 1985). The scale consists of five items rated on a seven-point scale from strongly disagree (1) to strongly agree (7). A total score is used to indicate the level of SWLS. The traditional Chinese version of the SWLS was validated by Wu and Yao (2006). The Cronbach's *α* coefficients were 0.91, 0.91, and 0.91 in the pooled sample and in samples 1 and 2, respectively.

## Results

Table 1 presents the descriptive statistics of the research variables. We first performed a CFA to validate the association between the latent factor of the BRS and the latent factor of the CD-RISC. In the CFA model, the latent factor of the CD-RISC was indicated by five dimensions, and the latent factor of the BRS was indicated by six items since the BRS does not have subdimensions. We used dimensions, but not items, as the indicators for the CD-RISC to simplify its measurement structure because our aim is to examine the correlations between the latent factors of the BRS and the CD-RISC. Since the BRS has three positively worded items and three negatively worded items, a wording effect has been found in examining the factor structure of the BRS (Rodríguez-Rey *et al*., 2016; Tansey *et al*., 2016; Chmitorz *et al*., 2018; Fung, 2020). Following Chmitorz *et al*. (2018), who showed that a two-factor model, consisting of a general factor for all items and a method factor for negatively worded items, fit better than alternative models, we specified the same two-factor model for the BRS. The method factor could help to parcel out variances of the negative wording effect when we extract and examine latent factors of the BRS and the CD-RISC.

**Table 1.** Descriptive statistics and Pearson's correlations among all variables

Variable	*M*	s.d.	1	2	3	4
Pooled sample						
1. CD-RISC	62.36	12.85	–			
2. BRS	3.18	0.60	0.63**	–		
3. BDI-II	10.34	8.41	−0.52**	−0.51**		
4. SWLS	21.37	6.46	0.54**	0.43**	−0.55**	–
Sample 1						
1. CD-RISC	62.08	13.72	–			
2. BRS	3.11	0.63	0.65**	–		
3. BDI-II	11.51	9.51	−0.49**	−0.49**		
4. SWLS	20.79	6.57	0.45**	0.39**	−0.48**	–
Sample 2						
1. CD-RISC	62.59	12.11	–			
2. BRS	3.24	0.57	0.62**	–		
3. BDI-II	9.38	7.26	−0.55**	−0.52**	–	
4. SWLS	21.85	6.34	0.62**	0.46**	−0.63**	–

*Notes*: *N* = 448 for the pooled sample; *N* = 202 for study 1; *N* = 246 for study 2. **p < 0.01.

**Fig. 1.**
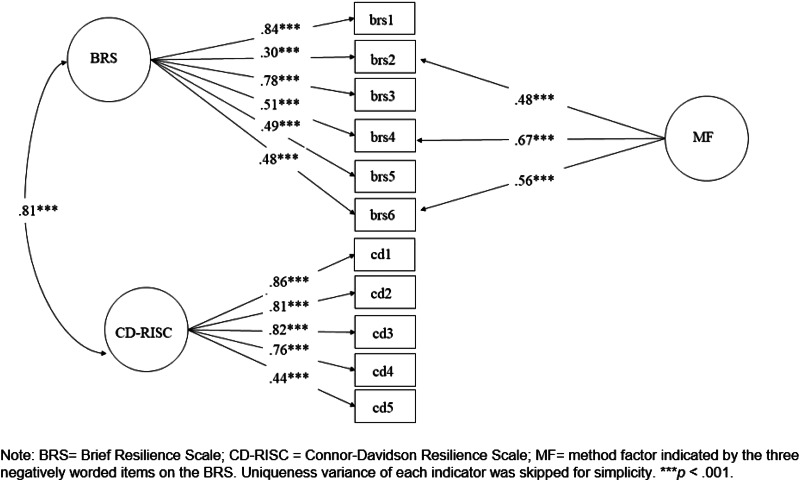
Standardized factor loadings from confirmatory factor analysis in the pooled sample.

We estimated the model using Mplus software, version 7.3, with the maximum likelihood estimator (Muthén and Muthén, 2012). Figure 1 presents the standardized factor loadings of the hypothesized CFA model. The model was acceptable (χ^2^ = 120.96, df = 40; CFI = 0.96, TLI = 0.95, RMSEA = 0.067, SRMR = 0.038). All of the estimates in the model were significant at *p* < 0.001. The latent factor of the BRS was positively related to the latent factor of the CD-RISC (*γ* = 0.81, *p* < 0.001). We also examined a model with a latent factor influencing all of the items of the BRS and the five dimensions of the CD-RISC and a method factor influencing the three negatively worded items of the BRS. This model (χ^2^ = 222.30, df = 41; CFI = 0.92, TLI = 0.89, RMSEA = 0.10, SRMR = 0.050) was worse than the model with two latent factors and one method factor (Δχ^2^ = 101.34, df = 1, *p* < 0.001).

To examine whether the BRS and the CD-RISC have unique effects in predicting the BDI-II and the SWLS, we performed regression analysis and present the results in Table 2. We found that both the BRS and CD-RISC negatively predicted scores on the BDI-II (*β* = −0.30, *p* < 0.001; *β* = −0.33, *p* < 0.001) and positively predicted scores on the SWLS (*β* = 0.16, *p* < 0.001; *β* = 0.44, *p* < 0.001). We then performed hierarchical regression analysis to examine the incremental predictive effect of the two measures on the BDI-II and the SWSL. As reported in Table 3, the BRS explained additional variances of the BDI-II [Δ*R*^2^ = 0.06, *F*_(1, 445)_ = 36.61, *p* < 0.001] and the SWLS [Δ*R*^2^ = 0.02, *F*_(1, 445)_ = 9.52, *p* < 0.01] beyond the CD-RISC. We also found that the CD-RISC explained additional variances of the BDI-II [Δ*R*^2^ = 0.06, *F*_(1, 445)_ = 41.94, *p* < 0.001] and the SWLS [Δ*R*^2^ = 0.12, *F*_(1, 445)_ = 74.78, *p* < 0.001] beyond the BRS.

**Table 2.** Standardized coefficients of multiple regression in samples 1 and 2

IV/DV	Pooled sample (*N* = 448)		Sample 1 (*N* = 202)		Sample 2 (*N* = 246)	
	BDI-II	SWLS	BDI-II	SWLS	BDI-II	SWLS
CD-RISC	−0.33***	0.44***	−0.30***	0.34***	−0.37***	0.55***
BRS	−0.30***	0.16***	−0.30***	0.18*	−0.29***	0.13*
*R*^2^	0.32	0.31	0.30	0.22	0.35	0.40
*F*	105.89***	97.61***	41.77***	28.48***	66.65***	80.45***

*p < 0.05; **p < 0.01; ***p < 0.001. (IV: Independent variables; DV: Dependent variables).

**Table 3.** *R*^2^ change in hierarchical regression analysis

IV/DV	Pooled sample (*N* = 448)		Sample 1 (*N* = 202)		Sample 2 (*N* = 246)	
	BDI-II	SWLS	BDI-II	SWLS	BDI-II	SWLS
Step 1: CD-RISC	0.27***	0.29***	0.24***	0.21***	0.30***	0.39***
Step 2: BRS	0.06***	0.02**	0.05***	0.02*	0.05***	0.01*
						
Step 1: BRS	0.26***	0.19***	0.24***	0.16***	0.27***	0.21***
Step 2: CD-RISC	0.06***	0.12***	0.05***	0.07***	0.09***	0.18***

*p < 0.05; **p < 0.01; ***p < 0.001. (IV: Independent variables; DV: Dependent variables).

**Fig. 2.**
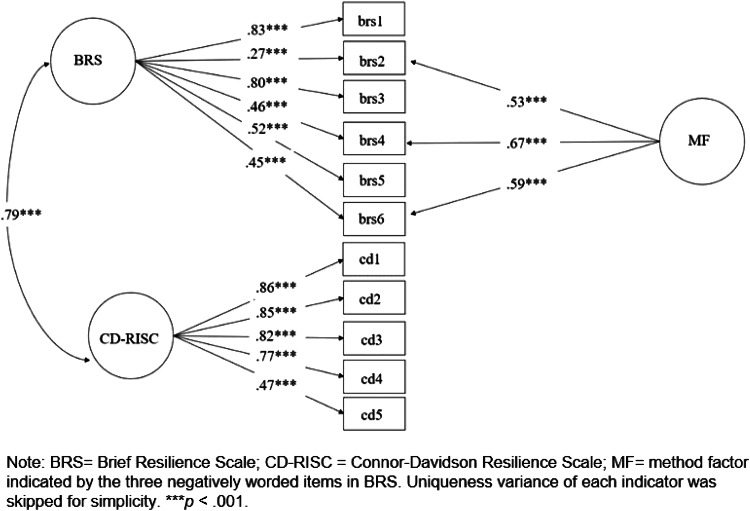
Standardized factor loadings from confirmatory factor analysis in sample 1.

**Fig. 3.**
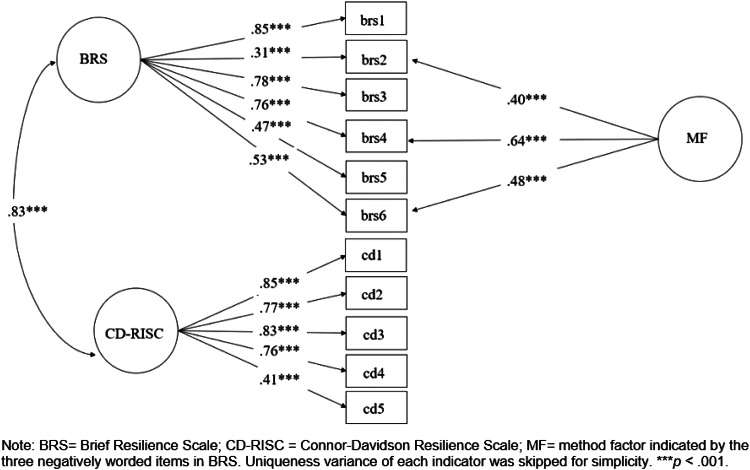
Standardized factor loadings from confirmatory factor analysis in sample 2.

For cross-validation purposes, we also performed the same analysis using samples 1 and 2 separately. Figures 2 and 3 present standardized factor loadings of the hypothesized CFA model in each sample. In sample 1, the model was acceptable (χ^2^ = 94.09, df = 40; CFI = 0.95, TLI = 0.93, RMSEA = 0.082, SRMR = 0.054). All of the estimates in the model were significant at *p* < 0.001. The latent factor of the BRS was positively related to the latent factor of the CD-RISC (γ = 0.79, *p* < 0.001). We also examined a model with a latent factor influencing all items of the BRS and the five dimensions of the CD-RISC and a method factor influencing the three negatively worded items of the BRS. This model (χ^2^ = 145.84, df = 41; CFI = 0.90, TLI = 0.87, RMSEA = 0.11, SRMR = 0.060) was worse than the model with two latent factors and one method factor (Δχ^2^ = 51.75, df = 1, *p* < 0.001).

In sample 2, the model with two latent factors and one method was also acceptable (χ^2^ = 88.04, df = 40; CFI = 0.96, TLI = 0.95, RMSEA = 0.07, SRMR = 0.042). All of the factor loadings were significant (*p* < 0.001). The latent factor of the BRS was also positively related to the latent factor of the CD-RISC (*γ* = 0.83, *p* < 0.001). We also examined a model with only a latent factor for all indicators and a method factor influencing the three negatively worded items. This model (χ^2^ = 140.04, df = 41; CFI = 0.92, TLI = 0.89, RMSEA = 0.10, SRMR = 0.055) was worse than the model with two latent factors and one method factor (Δχ^2^ = 52, df = 1, *p* < 0.001). We also performed an analysis to examine whether factor loadings obtained in samples 1 and 2 are invariant or not. Using a two-group analysis approach, we firstly estimated the same CFA model in samples 1 and 2 simultaneously (χ^2^ = 182.13, df = 78; CFI = 0.95, TLI = 0.94, RMSEA = 0.077, SRMR = 0.048) and estimated a model imposing equality of factor loadings across the two samples (χ^2^ = 189.59, df = 88; CFI = 0.96, TLI = 0.94, RMSEA = 0.072, SRMR = 0.055). The two models were not significant (Δχ^2^ = 7.46, df = 10, *p* = 0.68), suggesting invariance of factor loadings across the two samples.

Regarding regression analysis, in sample 1, we found that both the BRS and CD-RISC negatively predicted scores on the BDI-II (*β* = −0.30, *p* < 0.001; *β* = −0.30, *p* < 0.001) and positively predicted scores on the SWLS (*β* = 0.18, *p* < 0.05; *β* = 0.34, *p* < 0.001). As reported in Table 3, we found that the BRS explained additional variances of the BDI-II [Δ*R*^2^ = 0.05, *F*_(1, 199)_ = 14.55, *p* < 0.001] and the SWLS [Δ*R*^2^ = 0.02, *F*_(1, 199)_ = 4.59, *p* < 0.05] beyond the CD-RISC. We also found that the CD-RISC explained additional variances of the BDI-II [Δ*R*^2^ = 0.05, *F*_(1, 199)_ = 15.13, *p* < 0.001] and the SWLS [Δ*R*^2^ = 0.07, *F*_(1, 199)_ = 17.22, *p* < 0.001] beyond the BRS.

In sample 2, both the BRS and CD-RISC also negatively predicted scores on the BDI-II (*β* = −0.29, *p* < 0.001; *β* = −0.37, *p* < 0.001) and positively predicted scores on the SWLS (*β* = 0.13, *p* < 0.05; *β* = 0.55, *p* < 0.001). Similarly, the BRS explained additional variances of the BDI-II [Δ*R*^2^ = 0.05, *F*_(1, 243)_ = 19.26, *p* < 0.001] and the SWLS [Δ*R*^2^ = 0.01, *F*_(1, 243)_ = 4.01, *p* < 0.05] beyond the CD-RISC. We also found that the CD-RISC explained additional variances of the BDI-II [Δ*R*^2^ = 0.09, *F*_(1, 243)_ = 32.49, *p* < 0.001] and the SWLS [Δ*R*^2^ = 0.18, *F*_(1, 243)_ = 74.38, *p* < 0.001] beyond the BRS.

## Discussion

Across analysis using different samples, we found that the CD-RISC and BRS, although highly related, have unique predictive effects on measures of depression (i.e. BDI-II) and life satisfaction (i.e. SWLS). These findings suggest that the CD-RISC and BRS capture different aspects of resilience, indicating the need to differentiate resource-based resilience from belief-based resilience as two different concepts. From a theoretical perspective, such differentiation could help to expand the understanding of resilience. For example, if we consider resilience as a self-regulation process in which individuals undertake actions to recover from and adapt to adversity, then individuals will require resources to enable them to recover and a strong belief to motivate themselves and be persistent. From this perspective, the CD-RISC and BRS capture two elements that are essential to sustaining a resilience process. As such, our study not only simply demonstrates the differences between the CD-RISC and the BRS at a psychometric level but also indicates a potential theoretical implication by differentiating their conceptual differences.

This potential theoretical implication thus has implications for future studies of resilience and well-being. As mentioned earlier, studies have rarely used both the CD-RISC and the BRS at the same time to examine the role of resilience in individual well-being due to the lack of examination and acknowledgment of their differences. By showing that the CD-RISC and the BRS are two different concepts that potentially play different roles in driving a resilience process, our study suggests the need to further investigate the relationship between the CD-RISC and the BRS and their different functions in supporting individual well-being in a resilience process. In other words, only using either the CD-RISC or the BRS to indicate resilience could prevent us from depicting the full picture of resilience and its role in protecting individual well-being.

Practically speaking, our study suggests that the strengthening of resilience resources captured by the CD-RISC and the boosting of a belief in resilience emphasized by the BRS could be two different approaches to promoting individual well-being. While studies using either the CD-RISC or the BRS could only emphasize one approach rather than another, by examining the predictive effects of the two measurements on individual well-being simultaneously, our study suggests that these two approaches can be complementary to each other in contributing to individual well-being, such as greater life satisfaction and lower depression, at least among undergraduate students. Of course, this practical implication should be corroborated in intervention studies.

Finally, two limitations should be noted. First, we only used samples of undergraduate students from Taiwan. Whether the findings can be generalized to samples from other countries should be further examined. Second, we only included measurements of depression and life satisfaction as the two outcomes. Future studies should investigate whether the BRS and the CD-RISC also have unique predictive effects on other well-being or health-related outcomes. Future studies are also encouraged to explore when the BRS or the CD-RISC will become more important than the other in helping individuals to overcome and adapt to disruptions or stressful events. In other words, instead of investigating their differences with respect to resilient outcomes, identifying moderators or boundary conditions could also be essential to clarifying the differences between the BRS and the CD-RISC in capturing different aspects of resilience.

## Conclusions

In this study, we provide evidence suggesting the need to clarify the differences between the BRS and the CD-RISC, two widely used measurements of individual resilience. The statistical results were in line with our hypothesis showing that two instruments, despite being highly correlated, have unique predictive effects on depression and life satisfaction. Theoretically speaking, the uniqueness of the two instruments may result from the different conceptualization of resilience. While both measurements were developed to measure individual resilience, the CD-RISC focuses on available resources in multiple aspects that help individuals recover from and adapt to disruptions, and the BRS solely focuses on one's ability to bounce back from adversity, both of which are important in a self-regulatory resilience process. For future studies on resilience and well-being, researchers should pay closer attention to the differences between the two measurements and choose the best fit based on their research focus in order to be more precise with resilience measurement. To further unpack the differences between the CD-RISC and the BRS, future studies are encouraged to examine their different functions in shaping individual well-being from a self-regulatory perspective.

## Impact statements

Resilience is the ability to cope with daily stress and bounce back from adversity. Resilience is a crucial characteristic that directly impacts one's well-being. For example, it has been found negatively correlated with negative well-being indicators such as depression and anxiety while being positively correlated with positive well-being indicators including optimism, life satisfaction, and peace of mind. In this study, we examined the difference between two widely used measurements for resilience – the Brief Resilience Scale (BRS) and the Connor–Davidson Resilience Scale (CD-RISC). Our study found that the two scales capture different aspects of resilience and both have unique predictive effect on depression and life satisfaction. Our study brings light to knowledge about different aspects of resilience, which informs two approaches to building resilience.

## Data

All data generated or analyzed in this study are included in this published article.
